# Phenylalanine-Free Protein Substitutes for Next-Generation PKU Diets: Amino-Acid Roles, Transport, Absorption, and Formulation

**DOI:** 10.3390/foods15213944

**Published:** 2026-11-03

**Authors:** Dóra Kiss, Nevien Elhawat, Tarek Alshaal, Balázs Ásványi, Diána Szilva, Péter Hajdú, Attila Kiss

**Affiliations:** 1 Department of Food Biotechnology, Albert Kázmér Mosonmagyaróvár Faculty, Széchenyi István University, H-9200 Mosonmagyarovar, Hungary; kiss.dora@ga.sze.hu (D.K.); szilva.diana.margareta@sze.hu (D.S.); hajdu.peter@ga.sze.hu (P.H.); kiss.attila.peter@sze.hu (A.K.); 2 Albert Kázmér Faculty of Agricultural and Food Sciences, Széchenyi István University, H-9200 Mosonmagyarovar, Hungary; asvanyi.balazs@sze.hu

**Keywords:** Phenylketonuria, phenylalanine, protein substitutes, free amino acids, large neutral amino acids, intestinal absorption, blood–brain barrier, medical foods

## Abstract

Phenylketonuria (PKU) requires lifelong control of blood phenylalanine (Phe), but reliance on specialized protein substitutes varies with dietary Phe tolerance and response to pharmacotherapy. Sapropterin may permit dietary liberalization in responsive individuals, whereas successful pegvaliase enzyme substitution therapy may substantially reduce or eliminate the need for Phe-free protein substitutes. Conventional free-amino-acid formulations are nutritionally effective but may produce rapid plasma amino-acid peaks, increased oxidation, gastrointestinal intolerance, and treatment fatigue. Alternative systems, including casein glycomacropeptide (CGMP), oligopeptides, engineered low-phenylalanine proteins and controlled-release amino acids, may improve physiological utilization, but their relative metabolic and technological advantages remain uncertain. This narrative review qualitatively discusses the cited literature on amino-acid metabolism, intestinal absorption, tissue utilization, large-neutral-amino-acid transport, blood–brain barrier competition, nitrogen retention, safety, and food-formulation performance. Building on previous reviews, this article connects amino-acid roles, delivery-system characteristics, and analytical and clinical validation needs in a formulation-oriented conceptual framework. Its contribution is an integrated basis for defining product-development questions and corresponding endpoints, rather than a new biological finding or a validated prioritization method. The established nutritional importance of tyrosine in phenylalanine hydroxylase (PAH) deficiency is distinguished from unvalidated hypotheses for selectively enriching tryptophan or branched-chain amino acids. These distinctions describe nutritional roles and research needs rather than a quantitatively derived ranking. All indispensable amino acids remain necessary for nutritional adequacy, and rapid absorption is not a sufficient indicator of superior utilization. Future substitutes should combine balanced composition, controlled delivery, sensory acceptability, and rigorous clinical validation.

## 1. Introduction

Phenylketonuria (PKU) is a well-characterized inherited metabolic disease, usually caused by deficient activity of phenylalanine hydroxylase (PAH), the enzyme that converts phenylalanine (Phe) to tyrosine (Tyr). Without effective treatment, elevated Phe concentrations can cause neurotoxicity and severe cognitive impairment. Dietary treatment has traditionally relied on Phe restriction, with much of the protein requirement supplied by specially formulated protein substitutes [1]. These include free-amino-acid mixtures, glycomacropeptide (GMP)-based products, and extended-release preparations; large neutral amino-acid (LNAA) supplements provide another approach. These strategies differ in their metabolic effects and implications for daily dietary management [2,3]. Recent European guidelines emphasize individualizing treatment [1].

Impaired Phe metabolism leads to accumulation and potential neurological injury [1]. For patients requiring dietary treatment, management combines restriction of Phe-containing natural protein with Phe-free amino-acid preparations supplemented with Tyr, vitamins and minerals [3]. Reliance on manufactured substitutes makes micronutrient adequacy an important consideration. Reviews have reported deficiencies in zinc, selenium, iron, vitamin B12 and, in some cases, folate, although these findings cannot be explained by dietary intake alone [4].

**Figure 1:**
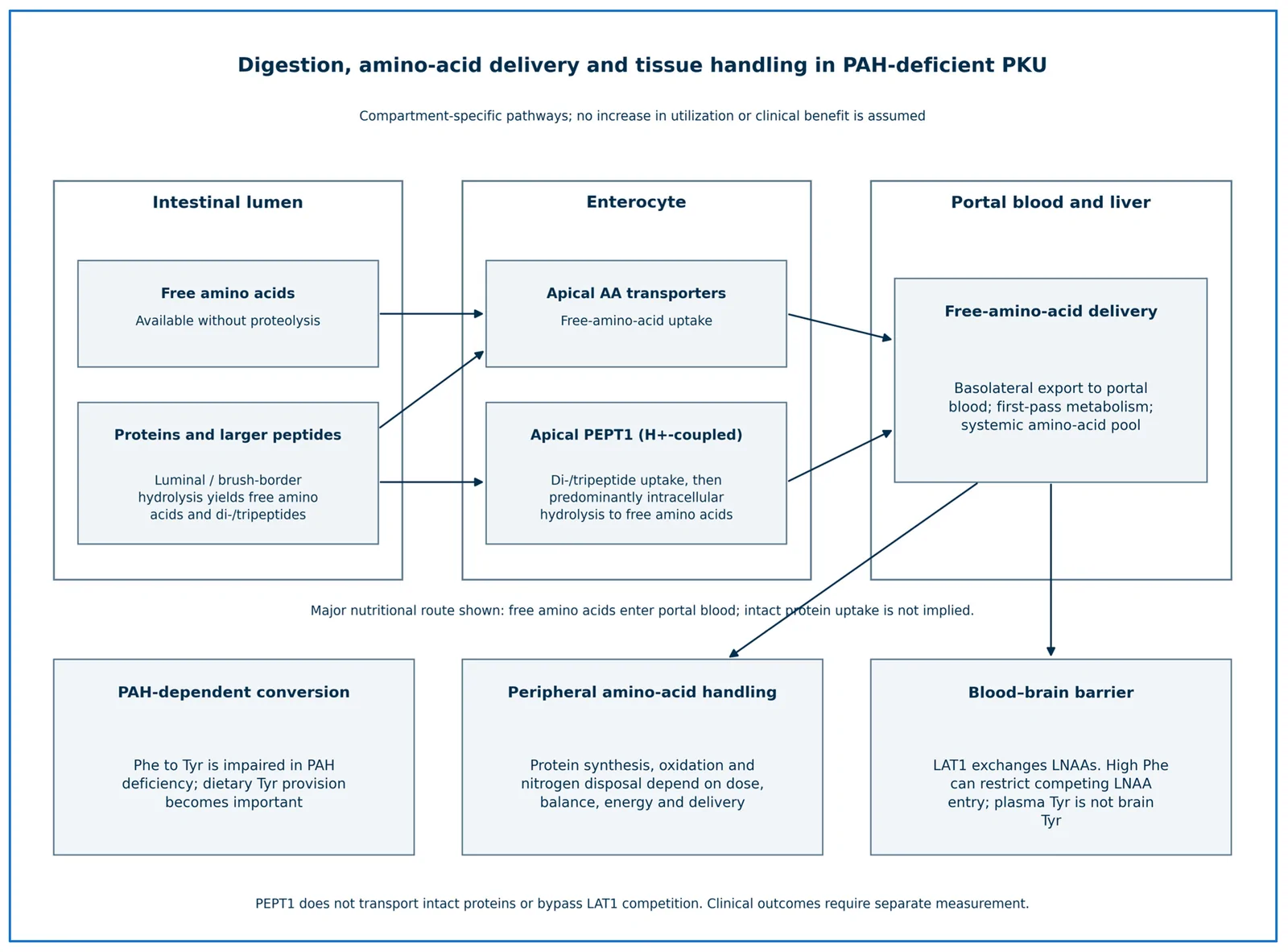
Major nutritional pathways of protein-substitute digestion and amino-acid handling in PAH-deficient PKU. Luminal and brush-border hydrolysis produces free amino acids and di-/tripeptides. Free amino acids use apical amino-acid transporters; PEPT1 mediates H+-coupled di-/tripeptide uptake, followed predominantly by intracellular hydrolysis. Basolateral export supplies free amino acids to portal blood before systemic distribution. Intact proteins are not PEPT1 substrates. PAH-dependent Phe-to-Tyr conversion and LAT1-mediated blood–brain barrier transport are distinct processes.

Free-amino-acid mixtures remain the most well-established format for PKU protein substitutes, but their rapid gastrointestinal availability, intense taste, high osmolality, and gastrointestinal burden create substantial food-formulation challenges [5]. CGMP, peptide-based systems, engineered low-phenylalanine proteins, encapsulated amino acids, and hybrid matrices have therefore emerged as potential alternatives. These systems differ in molecular structure, digestibility, peptide transport, gastric emptying, plasma amino-acid appearance, sensory quality, stability, and manufacturing feasibility [6,7]. Figure 1 outlines the links between substitute composition, gastrointestinal handling, tissue availability, and clinical outcomes that organize the subsequent discussion.

Previous reviews provide an important foundation for this topic. Daly et al. describe the historical evolution of PKU protein substitutes, including changes in composition, presentation, and delivery [8], while Keskin et al. review medical nutrition approaches and the efficacy and safety considerations of newer treatment and substitute strategies [9]. The present review builds on these accounts and shares several of their themes; it does not claim that amino-acid balance, Tyr provision, peptide delivery, or treatment acceptability are newly recognized. In particular, the nutritional importance of Tyr in PAH deficiency is established knowledge, not a novel finding of this review [1,8,9].

**Figure 2:**
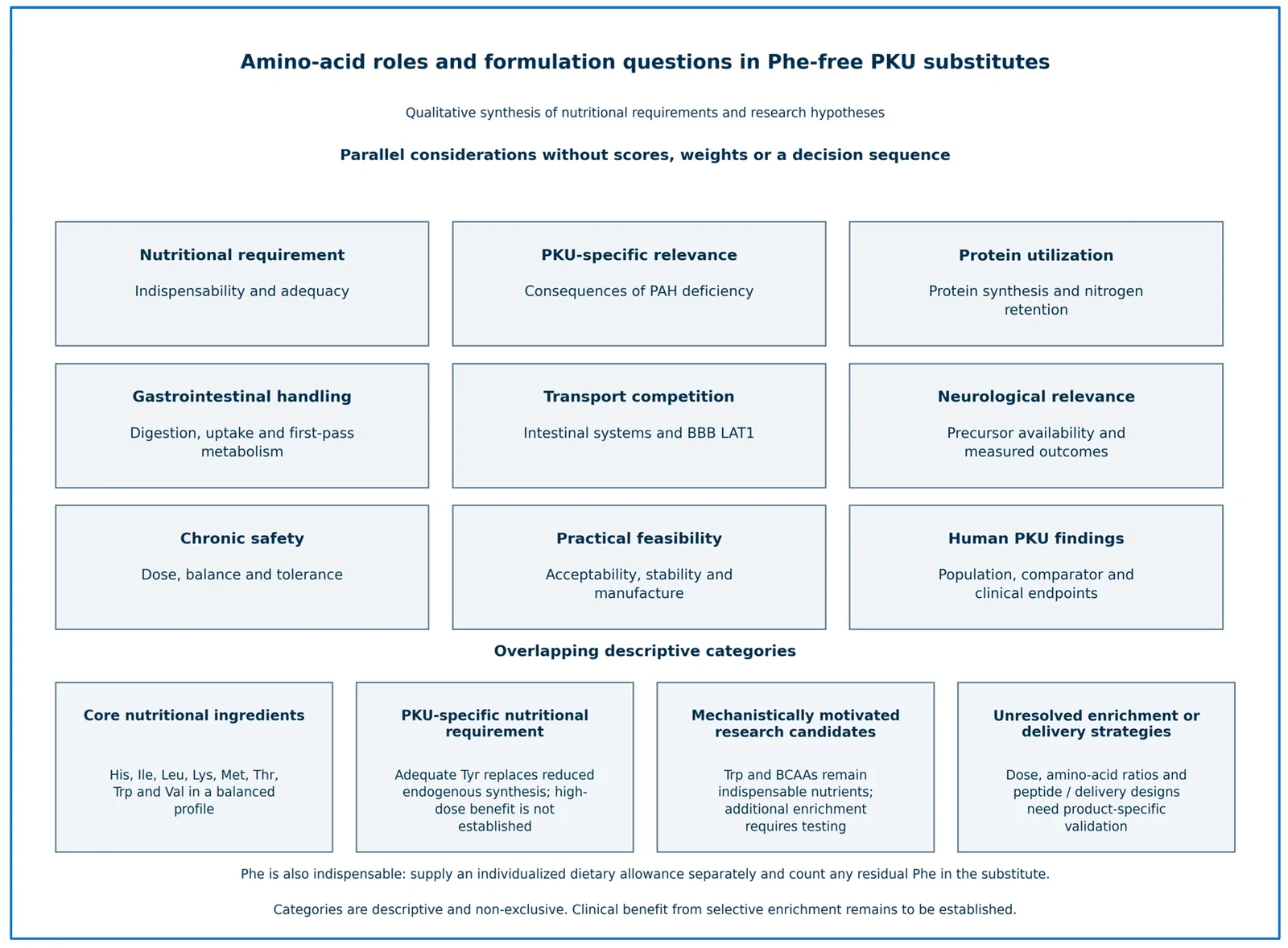
Nutritional roles and research questions for amino acids in Phe-free PKU protein substitutes. Core nutritional ingredients include the eight non-Phe indispensable amino acids, including Trp and all three BCAAs; Tyr has an additional PKU-specific nutritional requirement because endogenous synthesis is reduced. Phe remains indispensable and requires an individualized dietary allowance, including any residual Phe supplied by the substitute. Adequate nutritional inclusion is distinct from selective enrichment, whose clinical benefit requires demonstration. The nine domains are parallel considerations without numerical scores, weights, or a decision sequence.

**Figure 3:**
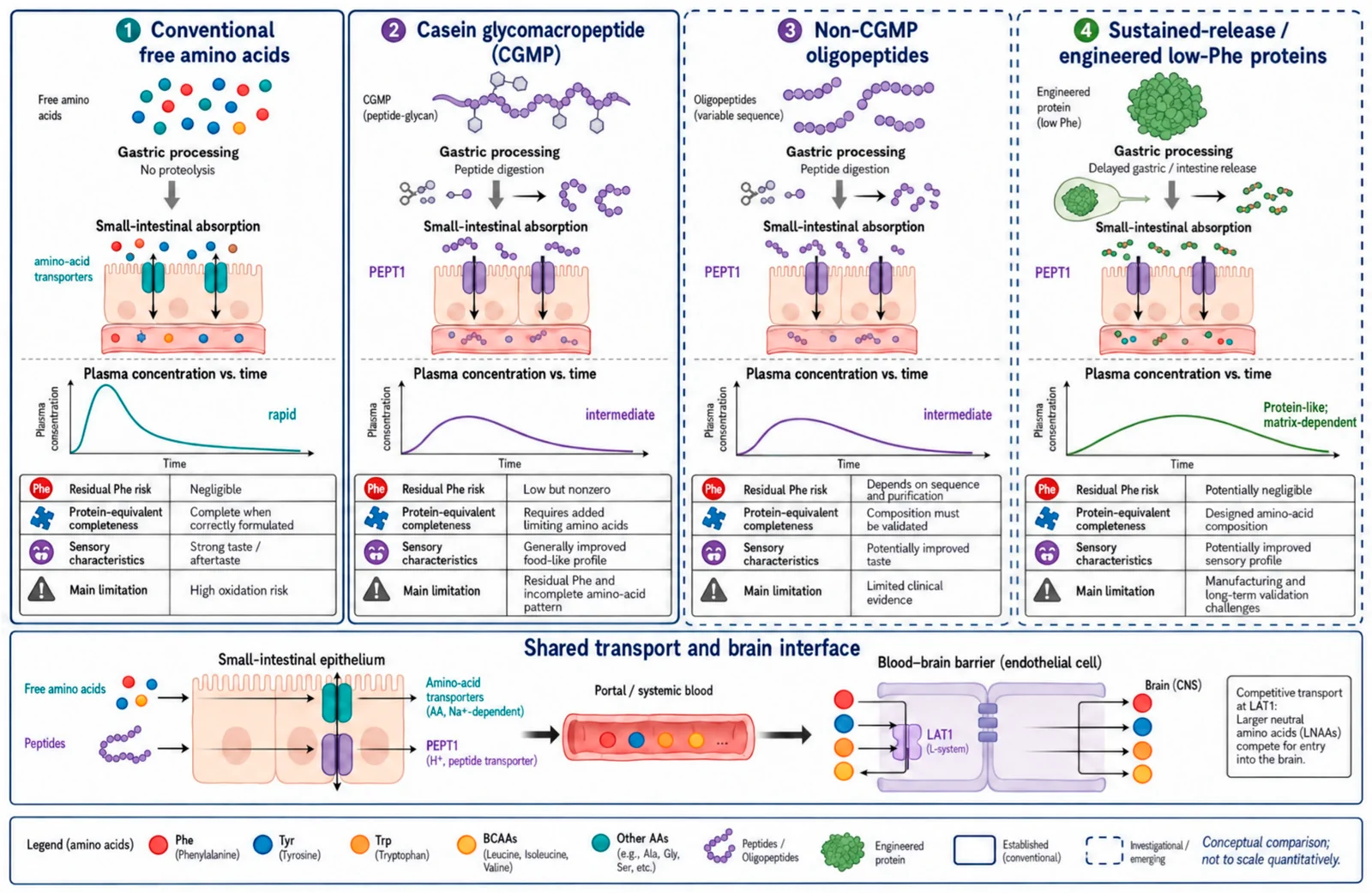
Comparison of digestive routes, kinetic interpretation, and compositional constraints across PKU protein-substitute systems. CGMP and other larger peptide or protein structures require digestion; PEPT1 takes up di-/tripeptides, while free amino acids use amino-acid transporters. Engineered low-Phe proteins and modified-release amino acids are separate strategies. A low-Phe sequence does not establish prolonged release, and a peptide fraction does not establish slower plasma appearance.

The intended additional contribution is a formulation-oriented synthesis that connects amino-acid roles with delivery-system properties and the measurements needed to evaluate a proposed product. Figure 2 distinguishes obligatory nutritional inclusion from selective-enrichment hypotheses, while Figure 3 and Table 1A,B separately compare delivery systems; Table 2 also presents the distinction between nutritional inclusion and selective enrichment. Section 7 and Section 8 translate these distinctions into analytical specifications and clinical research questions, including total-Phe exposure, nitrogen utilization, treatment-dependent substitute requirements, and patient-reported burden. This organization is intended to help researchers specify why an ingredient or delivery approach is being proposed and which outcomes would test its value. It is a conceptual aid to study and product design, not a validated ranking or evidence of clinical superiority.

### Review Scope and Interpretive Approach

This article is a narrative review of amino-acid nutrition, transport, metabolism, and formulation approaches relevant to PKU protein substitutes. The synthesis draws on clinical guidelines, human intervention and physiological studies, animal and in vitro studies, and published reviews cited throughout the article. It is not presented as a systematic review: literature coverage is not claimed to be exhaustive, and no reproducible database search strategy, search date range, or prespecified eligibility criteria are reported. No formal risk-of-bias assessments or certainty-of-evidence grades are assigned in this review. Human PKU findings are distinguished from findings in other populations and from preclinical or mechanistic observations. Descriptions of study findings and limitations are narrative interpretations of the cited literature, rather than standardized appraisal results.

The narrative approach may introduce selection and citation bias, and relevant studies may have been missed. Differences in study populations, formulations, comparators, and outcomes further limit comparisons. Consequently, the qualitative categories in Table 2 and Figure 2 should be interpreted as a synthesis of nutritional roles and research questions, rather than as formally graded evidence or comparative efficacy conclusions.

## 2. PKU-Specific Nutritional Constraints Relevant to Formulation

The physiological constraints relevant to formulation are summarized below. Section 3 examines digestion, absorption, and systemic handling; Section 4 compares amino-acid-specific roles; Section 5 integrates these findings into formulation questions.

The liver is the primary site of PAH expression and the initial site of the biochemical defect in PKU. Reduced hepatic Phe oxidation distinguishes this disease-specific constraint from the formulation-dependent handling of other amino acids discussed in Section 3 [7,10].

Whole-body protein turnover may remain within the reference range in cases of early-treated individuals receiving adequate energy and protein equivalents. Earlier pilot studies have found no consistent evidence of universal suppression of whole-body protein synthesis or increased whole-body catabolism due to PKU when dietary treatment is nutritionally adequate. This observation is important because it separates the direct metabolic consequences of hyperphenylalaninemia from the secondary effects of an artificial dietary pattern. Inadequate total energy, incomplete amino-acid delivery, long intervals between substitute doses, and poor adherence may generate negative nitrogen balance even when the underlying PAH defect is well controlled. Conversely, a complete substitute can maintain whole-body turnover despite persistent differences in plasma amino-acid concentrations [11,12].

PKU stems primarily from insufficient phenylalanine hydroxylase activity, limiting conversion of phenylalanine to Tyr and increasing dependence on dietary management. For patients who continue to require Phe-restricted dietary management, protein-substitute design entails three linked requirements: phenylalanine exposure must remain sufficiently low, Tyr must be supplied in adequate amounts, and all the other indispensable amino acids must be delivered in a balanced protein-equivalent pattern. The formulation objective is therefore not simply phenylalanine removal, but nutritional completeness together with predictable gastrointestinal delivery and acceptable food properties [1,13].

### 2.1. Protein Quality, Nitrogen Balance, and Amino Acid Utilization

Nitrogen balance reflects the relationship between nitrogen intake and nitrogen losses through urine, feces, skin, and other routes. In the case of PKU, the principal nutritional challenge is not only to provide the body with a proper amount of total nitrogen, but also to ensure a complete amino-acid pattern in a form that can be incorporated efficiently into body proteins. When the concentration of one indispensable amino acid is limited, the utilization of the remaining amino acids is constrained, and surplus nitrogen is directed toward deamination and urea synthesis. This principle is especially relevant to medical foods because their free-amino-acid profile can be precisely engineered, but an imbalance of one or more amino acids may reduce the effectiveness of the entire protein utilization [14].

Energy intake plays a central role in tailoring the nitrogen economy. When carbohydrate or fat intake is inadequate, amino-acid carbon skeletons are diverted toward energy production, increasing nitrogen disposal and reducing protein accretion. This relationship is particularly important in PKU, since low-protein diets may contain substantially variable carbohydrate contents. The impacts of a substitute cannot be evaluated independently of the implied total dietary and energy patterns. Growth, infection, inflammation and exercise might further alter the balance between synthesis, breakdown and oxidation. Clinical guidance consequently emphasizes monitoring growth, body composition, biochemical status and dietary adequacy, rather than relying on protein-equivalent intake alone [1,15].

For next-generation substitutes, nitrogen retention should therefore be treated as a primary performance endpoint. Useful measurements include urinary urea nitrogen, whole-body protein turnover, tracer-based amino-acid oxidation, postprandial plasma kinetics, muscle protein synthesis and growth, as well as lean-mass outcomes. These endpoints may lead to distinguishing a distinct formulation that merely raises plasma amino-acid concentrations from one that improves physiological utilization.

### 2.2. Formulation Implications of PKU-Specific Metabolism

PKU-specific metabolism should be translated into measurable food-design criteria, rather than handled solely as a clinical description. Firstly, the product must provide all the indispensable amino acids in sufficient amounts to prevent limiting amino-acid effects during tissue protein synthesis. Secondly, total Phe exposure should be evaluated using the analytical and dose-based reporting approach in the subsection “Free and Total Phenylalanine Analysis and Batch Release.” Thirdly, the amino-acid profile should be in accordance with the physiological importance of Tyr and the potential transporter interactions of Tyr, Trp, and the branched-chain amino acids. Fourthly, the delivery matrix should be evaluated for its effects on gastric emptying, peptide digestion, intestinal transport, plasma amino-acid excursions, oxidation, and urea production. Finally, the formulation performance must include sensory acceptability, gastrointestinal tolerance, physicochemical stability, manufacturing reproducibility, and regulatory compliance [6]. This integrated perspective shifts the focus from isolated biochemical mechanisms toward the design of complete medical foods whose composition, structure, and processing jointly determine their nutritional performance. Previous studies show that nutritionally complete, adequately distributed protein substitutes can support protein metabolism in treated PKU. However, formulation-specific effects on nitrogen retention and tissue protein synthesis remain insufficiently studied [11,12].

### 2.3. Pharmacotherapy and Changing Requirements for Protein Substitutes

Sapropterin and pegvaliase alter the nutritional context in which protein substitutes are used, through different mechanisms. Sapropterin is a synthetic tetrahydrobiopterin cofactor that enhances residual PAH activity in responsive individuals; it is not enzyme substitution therapy. Increased Phe tolerance may permit more natural protein and a reduction in protein-substitute intake, but responsiveness and the extent of dietary liberalization vary. Diet and substitute provision therefore require individual reassessment rather than automatic withdrawal of Phe restriction [16].

Pegvaliase is a pegylated phenylalanine ammonia lyase that provides an alternative route for Phe degradation, independently of residual PAH activity; it does not restore PAH-mediated conversion of Phe to Tyr [17]. The pegvaliase nutrition-management guideline recommends maintaining dietary management until a biochemical response is established, then progressively increasing intact protein and reducing medical food according to response and nutritional adequacy. Some responders can discontinue Phe-free medical foods, whereas others continue partial substitution. Monitoring must address low blood Phe, Tyr status, and nutrient adequacy as fortified medical foods are reduced [15].

These treatment trajectories suggest different design targets. People who remain substantially dependent on dietary Phe restriction need nutritionally complete, acceptable low-Phe or Phe-free substitutes. Responsive individuals who still require partial substitution may benefit from smaller, adjustable protein-equivalent portions whose amino-acid and micronutrient contributions can be integrated with increasing natural-protein intake. For pegvaliase responders meeting protein requirements through an unrestricted diet, routine use of a complete Phe-free substitute is not necessarily the appropriate objective. Diet quality and any specific nutritional deficits become more relevant than universal replacement of dietary protein. These are formulation implications of treatment-dependent nutritional needs, rather than demonstrated advantages of a particular new product.

Accordingly, the amino-acid considerations in Section 5 and Table 2 apply primarily when a protein substitute remains necessary. Tyr provision remains relevant because pegvaliase does not restore endogenous Tyr synthesis through PAH, but adequate Tyr may be supplied by natural protein; this does not imply a universal requirement for isolated Tyr supplementation or a Tyr-enriched substitute [15,17]. Transport-competitive LNAA enrichment should likewise not be assumed to provide additional benefit when pharmacotherapy has already achieved Phe control. Product selection should reflect current Phe tolerance, actual dietary intake, treatment response, and nutritional monitoring, rather than PKU diagnosis or drug exposure alone.

## 3. Digestion, Intestinal Absorption and Systemic Handling of Protein-Substitute Ingredients

### 3.1. Intact Proteins, Hydrolysates, Oligopeptides and Free Amino Acids

The nutritional performance of a PKU protein substitute depends on both its chemical composition and food structure. Amino acids may be supplied as free molecules, dipeptides or tripeptides, hydrolysates, GMP, engineered proteins, or combinations of these forms. Molecular size, peptide sequence, degree of hydrolysis, protein matrix, particle structure, viscosity, gastric emptying, and release behavior determine how rapidly substrates become available for intestinal uptake. Consequently, products with identical total amino-acid content may exhibit different plasma kinetics, splanchnic extraction, oxidation rates, and tissue availability [5,7].

Free-amino-acid mixtures used in PKU mostly bypass gastric and pancreatic proteolysis. Their amino acids are immediately available for transport across the intestinal epithelium, often displaying an early plasma peak followed by a relatively rapid decline. This profile can be advantageous when rapid correction of an amino-acid deficit is required, but it may also increase intestinal and hepatic extraction, amino-acid oxidation, and urea formation when the administered dose exceeds the immediate anabolic capacity. Scheinin et al. evaluated a specific prolonged-release formulation in a randomized, single-dose, four-way crossover study completed by 30 healthy adults [13]. Against the composition-matched immediate-release reference, the study-defined pooled EAA endpoint, which also included Tyr and Arg, had a Cmax ratio of 0.726 (approximately 27% lower; *p* < 0.0001). This is a group-level result, not a 27% reduction for each amino acid. Table 1 reports mean (SD) Cmax values of 1.77 (0.253) versus 2.44 (0.368) mmol/L, but no confidence interval for the Cmax ratio. The EAA AUC0–300 min ratio was 0.890 (90% CI 0.865–0.915), within the prespecified 0.80–1.25 bioequivalence range. This indicates lower measured exposure within that range, rather than identical exposure. These product-specific findings do not establish absolute absorption, tissue incorporation, or sustained clinical benefit in PKU.

Hydrolysates occupy an intermediate position. They contain peptides of different molecular weights and therefore require additional brush-border digestion before systemic absorption. The extent of their absorption depends on the peptide size distribution, degree of hydrolysis, enzyme accessibility, matrix composition, and the relative proportion of peptide and free-amino-acid transport. Hydrolysates may provide a less abrupt amino-acid appearance than free amino acids, but they cannot be assumed to have intact-protein kinetics due to the fact that extensive hydrolysis might generate a rapidly absorbable fraction [18,19]. For PKU, the principal limitation of using hydrolysates is compositional control: protein hydrolysates derived from ordinary food proteins may retain Phe-containing peptides, and the residual Phe concentration can vary according to the concentration of the raw material and the applied processing conditions.

The use of oligopeptides and CGMP provides a distinct strategy, as amino acids are delivered initially in peptide-bound form. CGMP is naturally low in Phe; however, it is not nutritionally complete without supplementation with additional amino acids, particularly His, Leu, Met, Tyr and Trp. Human crossover data indicate that CGMP-based medical foods may support metabolic control and improve acceptability, but their clinical value depends on the amount of residual Phe, supplementation pattern, and total protein-equivalent intake [5,20]. Thus, the relevant comparison is not based on “protein versus amino acids” in general, but on the relationship between the peptide structure, amino-acid completeness, residual Phe, and postprandial handling.

### 3.2. Intestinal Transport Systems and Peptide-Mediated Absorption

Absorption mechanisms of intestinal amino acids involve multiple transporter families with overlapping substrate specificities and distinct apical or basolateral localization. Neutral amino acids are transported by systems such as B^0^ and L, whereas cationic amino acids, including Lys and arginine (Arg), are transported by systems b^0,+^, y^+^, and y^+^L. These systems are mediated by transporters including CAT-family proteins and heteromeric amino-acid transporters. Aromatic amino acids and BCAAs can share system L pathways, creating competition when luminal or plasma concentrations are high. The transporter environment is therefore central to PKU formulation, since Phe, Tyr, Trp, Leu, Ile, and Val are not absorbed independently [21,22,23].

Peptide-mediated absorption is dominated by PEPT1, the proton-coupled oligopeptide transporter encoded by SLC15A1. PEPT1 transports a broad range of dipeptides and tripeptides, allowing several amino acids to enter enterocytes in peptide-bound form. Intracellular peptidases then hydrolyze these peptides, and the resulting amino acids leave the enterocyte through basolateral transport systems. Peptide transport may increase the number of absorbable molecular species and reduce the direct competition among free amino acids at apical transporters [19,21]. However, PEPT1 transport does not guarantee slower or more efficient systemic delivery, as intracellular hydrolysis, basolateral export, portal extraction, and hepatic metabolism remain influential.

The distinction between intestinal and blood–brain barrier transport is essential. Intestinal amino-acid transport determines entry into the portal circulation, whereas LAT1-mediated transport at the blood–brain barrier determines the relative cerebral availability of Phe and other LNAAs. Increased amounts of luminal Tyr or Leu do not necessarily result in proportional brain delivery, due to competition that may occur at several levels: apical intestinal uptake, plasma protein-free concentration, endothelial transport, and intracellular cerebral metabolism. LAT1 is a heteromeric transporter with broad specificity for large neutral amino acids, and its activity is influenced by substrate concentration, transporter expression, as well as the environment of the competing amino acids [24,25].

Recent experimental evidence indicates that protein-substitute composition might largely influence intestinal transporter expression and nutrient absorption beyond the amino-acid delivery itself. An in vitro study comparing the behavior of L-amino-acid and CGMP-amino-acid preparations pointed out distinct effects on intestinal nutrient transport and bacterial growth, including differences in LAT1 and PEPT1 regulation [26]. These observations remain mechanistic and might not establish clinical superiority; however, they support a broader formulation concept in which the food matrix may influence transporter biology, intestinal signaling, and microbiota-mediated nutrient handling.

For next-generation substitutes, transport must therefore be considered as a sequence of distinct factors and processes, rather than a single event. The relevant questions are whether a formulation provides a balanced entry into enterocytes, avoids excessive competition, preserves Tyr and Trp availability, maintains adequate delivery of indispensable amino acids, and produces a plasma pattern compatible with tissue utilization. Peptide-based delivery may be especially valuable when it provides compositional accuracy and controlled hydrolysis; however, residual Phe and incomplete amino-acid profiles remain decisive constraints.

### 3.3. Plasma Kinetics, Oxidation, Urea Production, and Tissue Availability

Plasma concentration–time profiles provide a practical description of the behavior of a protein substitute after ingestion, but they do not directly include tissue utilization. Immediate-release free-amino-acid mixtures typically trigger rapid increases in the concentrations of essential, branched-chain, and aromatic amino acids, followed by declining concentrations as amino acids are incorporated, oxidized, transported into tissues, or excreted. A high maximum concentration might indicate efficient absorption; however, it can also reflect immediate enhancement of the capacity for protein synthesis. The more informative parameters are the relationships between maximum concentration, exposure over time, plasma disappearance, oxidation, urinary nitrogen, and tissue incorporation [27,28].

Amino-acid oxidation is particularly important when the delivery pattern is rapid, or the amino-acid profile is unbalanced. Amino acids cannot be stored in a dedicated reserve pool; excessive amounts of amino groups are transferred or removed, and the resulting nitrogen is incorporated into urea. Repeated high-dose administration may therefore increase blood urea nitrogen and urinary nitrogen without improving protein accretion. Conversely, gradual delivery can maintain plasma availability over a longer period and may reduce the mismatch between absorption and tissue demand. Acute changes in urea, insulin, or glucose are indirect metabolic observations; they do not by themselves demonstrate improved nitrogen retention or tissue protein synthesis. The healthy-volunteer experiment summarized in Section 3.1 should therefore not be interpreted as clinical validation of improved protein utilization in PKU.

Systemic and cerebral availability require different interpretations. Human imaging links higher Phe exposure with reduced Tyr influx and cerebral protein synthesis [24], whereas plasma concentration–time studies measure circulating availability. These endpoints are complementary but cannot be treated as interchangeable evidence of tissue utilization. CGMP-based products illustrate why the physical presence of a peptide fraction does not by itself establish slower amino-acid appearance or superior utilization; supplemented CGMP can have plasma kinetics resembling a free-amino-acid product [20]. For non-CGMP systems, human studies of protein hydrolysates provide relevant but indirect evidence. Koopman et al. found faster digestion and absorption of hydrolyzed casein than intact casein in older men, without a statistically significant difference in the measured muscle protein synthesis response [29]. Deglaire et al. reported faster absorption and altered regional nitrogen handling after hydrolyzed casein, but similar whole-body postprandial nitrogen retention compared with intact casein in healthy adults [19]. These findings concern specific casein hydrolysates, not defined Phe-free oligopeptides or clinical efficacy in PKU. Candidate products therefore require characterization of peptide composition, free and total Phe, and direct evaluation of nitrogen utilization; plasma appearance alone is insufficient.

A clinically meaningful comparison of protein substitutes should therefore include their plasma concentration–time curves, areas under the curve, maximum concentrations, times to maximum concentration, amino-acid oxidation, urea production, urinary nitrogen, whole-body protein turnover and, where feasible, tissue-specific synthesis. The most promising formulation is not necessarily the one that generates the highest plasma peak; it is the one that provides adequate amino-acid exposure with minimal unnecessary oxidation, preserves the relative availability of Tyr and other LNAAs, supports protein accretion and remains acceptable for sustained use.

### 3.4. Integrated Gastrointestinal Handling of Food Matrices

Table 1A,B separate metabolic characteristics from practical product attributes. Across studies, differences in amino-acid composition, protein-equivalent dose, energy co-ingestion, and participant population limit attribution of an observed effect to delivery structure alone. The hydrolysate comparisons in Section 3.3 show why faster absorption cannot be equated with greater retention [19,30]; the clinical comparisons in Section 6 assess whether product-level differences translate into useful PKU outcomes. Sensory quality, processing and manufacturing are examined separately in Section 7.

## 4. Comparative Metabolism and Transport of PKU-Consistent Candidate Amino Acids

### 4.1. Tyrosine and Tryptophan: Conditional Requirements and Neurotransmitter Precursors

Tyr occupies a distinctive position in PKU, since the PAH reaction that normally produces Tyr from Phe is impaired. Tyr consequently shifts from being a nutritionally non-essential amino acid toward a conditionally essential nutrient. Tyr supports protein synthesis and neurotransmitter production. In PKU, the requirement for exogenous Tyr is determined by residual PAH activity, dietary Phe exposure, growth, illness, and the amount of Tyr supplied through natural protein and medical foods [1,12].

The transport findings summarized in Section 3.2 and Section 3.3 support a PKU-specific rationale for adequate Tyr provision [24]. They do not establish that isolated high-dose Tyr supplementation improves neurological outcomes; that question requires clinical endpoints beyond plasma Tyr elevation.

Trp is also an LNAA and competes with Phe for transport into the brain. Its metabolic importance extends beyond protein synthesis, as it is the precursor of serotonin, melatonin, and kynurenine-pathway metabolites. In PKU, a high Phe concentration can reduce the relative availability of Trp at the BBB, potentially contributing to altered serotonin synthesis, sleep regulation, mood, and executive function. LNAA supplementation has been associated with changes in the concentrations of melatonin and neurotransmitter-related biomarkers, although the clinical significance remains uncertain, and the number of direct trials on isolated Trp supplementation is limited [31,32].

### 4.2. Branched-Chain Amino Acids: Leucine, Isoleucine and Valine

Leu, Ile and Val are indispensable BCAAs required for the synthesis of tissue proteins and the maintenance of nitrogen balance. BCAAs contribute to peripheral protein metabolism, but excessive bolus delivery may increase plasma excursions and oxidation [33,34].

Leu has an additional signaling role. It activates the mTORC1 pathway through nutrient-sensing mechanisms and may stimulate translation in skeletal muscle. This makes Leu a plausible candidate for improving the anabolic efficiency of Phe-free protein substitutes, particularly in children, adults with low lean mass, and physically active patients. However, Leu alone cannot be responsible for the accomplishment of complete protein synthesis. This response still requires the simultaneous availability of all other indispensable amino acids. A Leu-enriched formulation may therefore fortify anabolic signaling without increasing the net protein deposition if another amino acid becomes limiting [35,36].

The signaling rationale does not establish a benefit from selective BCAA enrichment in PKU. Studies should distinguish muscle protein synthesis and nitrogen retention from plasma BCAA excursions, and assess effects on the wider LNAA balance before recommending enrichment [37].

### 4.3. Lysine, Threonine, Methionine and Histidine: Indispensable Nutritional Functions

Lys, Thr, Met, and His are indispensable amino acids; thus, they must be supplied in adequate amounts on a Phe-free diet as well. Their principal role is not direct competition with Phe, but the completion of the amino-acid pattern required for tissue protein synthesis. If one of these amino acids is limiting, the utilization and absorption of the other ones decrease, as translation cannot proceed efficiently during the synthesis ofproteins requiring the limiting building block. The result of this phenomenon is increased amino-acid oxidation and nitrogen excretion despite an apparently adequate total protein-equivalent intake [38].

Lys does not share the principal LAT1 pathway used by Phe, Tyr, Trp, and BCAAs; therefore, its importance in PKU substitutes is primarily the prevention of a limiting-amino-acid deficiency [39,40,41]. Thr and Met are indispensable components of a nutritionally complete substitute, but neither currently warrants PKU-specific enrichment beyond an age-appropriate balanced amino-acid pattern [1]. CGMP is naturally low in several indispensable amino acids, including His, Met, and Trp; therefore, it requires targeted supplementation to achieve a nutritionally complete pattern [5,20].

The conceptual framework distinguishes the obligatory nutritional inclusion of Lys, Thr, Met, and His from hypotheses for additional enrichment. A limiting concentration or patient-specific deficiency may justify adjustment, but no comparative therapeutic rank is assigned. A rational substitute therefore requires a complete profile of the indispensable amino acids, preceding any optional enrichment with transport-competitive or anabolic amino acids. Protein quality cannot be improved by increasing Tyr or Leu, while allowing Lys, Thr, Met, or His to become limiting.

### 4.4. Large Neutral Amino Acid Competition at the Intestine and Blood–Brain Barrier

The intestinal and blood–brain barrier mechanisms are described in Section 3.2. Their clinical relevance is assessed here by comparing the scope and limitations of LNAA studies.

LNAA supplementation has been developed around the hypothesis that increasing the concentration of non-Phe LNAAs might reduce the relative influx of Phe into the brain, while improving the availability of Tyr, Trp, and BCAAs. Early clinical studies reported reductions in the concentrations of Phe in plasma or brain, as well as changes in neurochemical markers; however, the trials were small and differed in composition, dose, background diet, and treatment adherence. A long-term study of a Phe-neutral LNAA medical food revealed the potential benefits for Phe control and treatment acceptability, but it did not establish that one specific LNAA ratio is superior to standard protein substitutes [31,42].

Competition is not selective for Phe. A high concentration of one non-Phe LNAA can reduce the transport of another one, and the outcome depends on affinity, concentration, transporter expression, and the relative abundance of all competing substrates. Increasing the amount of Leu, for example, may reduce Phe transport but may also influence Tyr or Trp entry. Similarly, increasing the concentration of Tyr may improve the Tyr:Phe ratio while altering the transport of other aromatic amino acids. This competitive network makes it inappropriate to infer therapeutic effectiveness from the concentration of a single amino acid in plasma. Previous research identifies Tyr and balanced LNAAs as the candidates most directly linked to PKU-specific metabolism and cerebral transport. The optimal amino-acid ratios for improving brain delivery and clinical outcomes remain unknown [24,43].

## 5. Qualitative Synthesis of Amino Acid Roles and Formulation Considerations

### 5.1. Scope and Interpretation of the Qualitative Framework

This section presents a conceptual framework for organizing the narrative literature on amino-acid roles in PKU protein-substitute design. It is not a reproducible multi-criteria scoring analysis. The nine domains in Figure 2 are prompts for discussion, not scored variables; no numerical scales, relative weights, aggregation rules, or candidate rankings were applied. Table 2 records descriptive categories, their biological rationales, the cited findings and limitations, and formulation implications. These categories represent the authors’ narrative interpretation of the cited literature, rather than the output of an independently validated classification procedure. Nutritional inclusion and selective enrichment are considered separately: adequate provision of indispensable amino acids is required, whereas enrichment above requirements needs additional justification. Individual amino acids, mixtures, and delivery platforms are not treated as interchangeable alternatives [1,24]. The practical purpose of the framework is to make the link between a proposal and its validation explicit. For example, adequate Tyr provision addresses an established nutritional requirement, whereas a claim for additional benefit from Tyr enrichment requires separate outcome data. Similarly, a slower-release formulation requires assessment of complete delivery and utilization rather than inference from a lower plasma peak alone. The framework organizes these questions; it neither establishes a new biological mechanism nor determines an optimal amino-acid dose or formulation.

The descriptive categories are defined by their purpose. “Core nutritional ingredients” identifies amino acids required to complete an indispensable-amino-acid profile; this includes Trp and all three BCAAs, irrespective of any additional research rationale. “PKU-specific nutritional requirement” identifies Tyr because impaired PAH-mediated synthesis increases reliance on exogenous supply, without establishing benefit from high-dose enrichment. “Mechanistically motivated research candidates” identifies selective-enrichment hypotheses related to transport competition, neurotransmitter precursors, or anabolic signaling; a plausible mechanism does not establish clinical efficacy. “Unresolved enrichment or delivery strategies” identifies proposals whose suitability remains dependent on dose, composition, delivery characteristics, or clinical validation. Category membership is non-exclusive and does not indicate an ordinal priority level. Safety, available human findings, and formulation feasibility qualify the discussion but are not numerically combined to assign a category or overall rank.

### 5.2. Nutritional Indispensability and Protein-Quality Considerations

The nutritional roles described in Section 4 imply that completeness must be assessed before optional enrichment. Product comparisons should report the complete amino-acid pattern and protein-equivalent dose; otherwise, a response to supplementation may reflect correction of a deficiency rather than a specific advantage of the enriched amino acid [1,12].

CGMP illustrates the distinction between an ingredient and a nutritionally complete product: its performance must be interpreted together with the free amino acids added to correct its composition [5,20]. This limits attribution of product-level outcomes to the peptide fraction alone.

### 5.3. PKU-Specific Metabolic and Neurological Relevance

PKU-specific relevance describes how an amino acid relates to the consequences of PAH deficiency in addition to its general nutritional functions. Tyr has a direct PKU-specific nutritional rationale, as the PAH reaction normally supplies Tyr from Phe. The resulting conditional Tyr dependence affects both protein synthesis and the production of catecholamines and thyroid hormones. In addition, high concentrations of Phe competitively reduce the extent of Tyr transport into the brain through LAT1. Human imaging studies have linked increased Phe exposure to reduced cerebral Tyr influx and lower cerebral protein-synthesis rates, providing direct mechanistic support for the nutritional relevance of Tyr [24].

Trp has a plausible PKU-specific functional rationale, although the clinical benefit of isolated enrichment remains uncertain. It is an indispensable LNAA and a precursor for serotonin, melatonin, and kynurenine metabolites. Increased amounts of Phe may lead to the reduction of the relative availability of Trp at the BBB, potentially affecting serotonin-related neurochemical pathways. However, the cited mechanistic findings do not establish the clinical benefit of isolated Trp supplementation in humans with PKU. In most of the LNAA-related studies, combined mixtures were used, preventing attribution of changes in mood, sleep, melatonin, or neuropsychological outcomes to Trp alone. Thus, Trp is a promising functional component, but may not be considered an agent of clinically established, independent therapy [31,32].

BCAAs may influence PKU through two mechanisms: they provide indispensable substrates for peripheral protein synthesis, and they compete with Phe for LNAA transport. Leu also activates mTORC1. However, the neurological effect of BCAA enrichment is difficult to predict, since BCAAs compete not only with Phe, but also with Tyr and Trp. An increase in the concentration of one LNAA may therefore improve its transport relationship, while worsening that of another one. Animal studies support the possibility that balanced LNAA supplementation reduces brain Phe and improves neurochemical abnormalities, but human evidence remains limited and formulation-specific [32,44].

Lys, Thr, Met, and His are discussed primarily in relation to nutritional completeness; the cited literature does not establish selective enrichment as a means of directly modifying Phe toxicity or BBB transport. Their absence or inadequacy, however, would compromise the biological value of the entire substitute. This distinction is fundamental: limited evidence for an additional PKU-specific therapeutic role does not imply low nutritional importance. It indicates that the amino acid is necessary for completeness, but currently no evidence supports the necessity of additional enrichment as a Phe-lowering or neuroprotective strategy [43].

In particular, outcomes after an LNAA mixture cannot identify the independent contribution of Trp or an optimal BCAA ratio. Differences in mixture composition, dose, background treatment and endpoints further constrain comparisons. Selective enrichment therefore requires a matched comparator and patient-relevant outcomes, while adequate nutritional inclusion remains necessary regardless of evidence for an additional therapeutic effect [31,42,43].

### 5.4. Absorption, Transporter Competition, Safety and Formulation Feasibility

Absorption and transporter behavior determine whether an amino acid delivered in a medical food reaches the tissue for which it was prioritized. Free amino acids are absorbed rapidly and may generate high plasma peaks, whereas CGMP, oligopeptides and prolonged-release systems may modify the time course of appearance. The single-dose healthy-volunteer findings summarized in Section 3.1 illustrate product-specific modification of plasma kinetics, without establishing improved utilization or clinical benefit in PKU. Similar technology has been used to mask taste and odor, as well as to extend intestinal release [7].

Transport competition is especially important for Tyr, Trp, and BCAAs because they share large neutral amino-acid transport systems. LAT1 at the BBB transports Phe, Tyr, Trp, and BCAAs, so the effect of increasing one substrate depends on the concentration of all the others. The intestinal transporter environment is also relevant because luminal competition can alter portal delivery before the amino acids reach the BBB. A formulation with a favorable plasma Phe:Tyr ratio may still provide limited cerebral benefit if the absolute concentrations, timing, and relative abundance of competing LNAAs remain unfavorable [24,25].

Safety is a separate criterion from mechanistic plausibility. Tyr, Trp, and BCAA enrichment may alter neurotransmitter metabolism, insulin responses, oxidation, urea production, gastrointestinal tolerance, and interactions among competing amino acids. Chronic exposure to a single enriched amino acid may also generate secondary imbalance if the total formulation is not adjusted. CGMP introduces an additional safety issue, as residual Phe contributes to the patient’s daily Phe allowance. Clinical CGMP studies have demonstrated feasibility, but residual Phe, incomplete amino-acid composition, and long-term nutritional consequences require product-specific monitoring [5,8].

Formulation feasibility includes handling matters such as solubility, taste, odor, stability, hygroscopicity, osmolality, processing tolerance, cost, and regulatory reproducibility. An amino acid with a strong metabolic rationale may be difficult to incorporate into an acceptable formulation if it is characterized by unacceptable taste or destabilizes the product. Conversely, a peptide or engineered protein may have favorable sensory properties but require sophisticated analytical methods to confirm the negligible concentration of Phe and the consistent digestibility. Recent food-science literature findings emphasize that medical-food innovation must integrate biochemical composition with sensory and manufacturing performance [10].

### 5.5. Qualitative Synthesis, Evidence Limitations and Formulation Context

Table 2 summarizes the nutritional roles, PKU-specific rationales, evidence limitations, and formulation implications of selected amino acids and LNAA-based strategies. Its categories are descriptive and non-exclusive: an amino acid may be indispensable for nutritional completeness while also having a mechanistic rationale for further investigation. The categories do not represent numerical scores, comparative treatment effects, or a validated ranking. Figure 2 presents the same non-exclusive categories as a conceptual map. “Core nutritional ingredients” denotes adequate provision of all indispensable amino acids. “Mechanistically motivated research candidates” denotes hypotheses for selective enrichment, including Trp and selected BCAAs, rather than a separate class of nutritionally optional amino acids. Trp, Leu, Ile, and Val remain core nutritional ingredients regardless of whether enrichment is investigated. Tyr is distinguished by its PKU-specific nutritional requirement; balanced LNAA mixtures are formulation strategies rather than individual amino acids.

**Table 2:** Qualitative synthesis of nutritional roles, PKU-specific rationales, and formulation implications of selected amino acids and LNAA strategies.

Descriptive Category	Amino Acid or Formulation	Unit Considered	PKU-Specific Rationale	Summary of Cited Findings and Limitations	Formulation Implication	References
PKU-specific nutritional requirement	Tyrosine (Tyr)	Individual amino acid	PAH deficiency reduces endogenous Tyr synthesis; Tyr supports protein synthesis and catecholamine production; high Phe may restrict cerebral Tyr transport.	Direct nutritional rationale; additional isolated Tyr raises plasma Tyr, but consistent neurocognitive or quality-of-life benefit is unproven.	Ensure adequate, physiologically distributed Tyr; avoid interpreting plasma elevation alone as improved cerebral availability.	[12,24,45]
Core nutritional ingredients	Lysine (Lys), threonine (Thr), methionine (Met), histidine (His), isoleucine (Ile), and valine (Val)	Individual amino acids (listed together for brevity)	Indispensable amino acids required for complete protein-equivalent delivery, tissue protein synthesis, and nitrogen retention. Ile and Val are individual indispensable BCAAs, considered here within the complete amino-acid profile rather than as a separate formulation.	Adequate provision is nutritionally necessary; the cited literature does not establish an independent PKU-specific benefit from selectively enriching Ile or Val above requirements.	Prevent limiting-amino-acid effects before considering selective enrichment; supply Ile and Val in balanced proportions with Leu and the other indispensable amino acids.	[1,9,38,41]
Mechanistically motivated research candidates	Tryptophan (Trp)	Individual amino acid	LNAA and precursor of serotonin, melatonin, and kynurenine metabolites; competes with Phe for cerebral transport.	Mechanistic rationale: the cited literature does not establish clinical benefit from isolated Trp supplementation in PKU.	Consider primarily within balanced LNAA formulations; monitor competition and safety.	[31,32]
Mechanistically motivated research candidates	Leucine (Leu)	Individual amino acid	Indispensable BCAA; activates mTORC1 and contributes to peripheral protein synthesis; also participates in LNAA competition.	In healthy young adults, a higher Leu proportion in an essential-amino-acid mixture increased anabolic signaling without greater net muscle protein anabolism [33]. The cited human studies do not establish clinical benefit from selective Leu enrichment in PKU.	Maintain balanced BCAA delivery; avoid selective enrichment without complete amino-acid support.	[35,36,40]
Mechanistically motivated research candidates	Balanced non-Phe LNAA formulation	Amino-acid group/formulation	May alter relative Phe transport at the intestine and BBB while supporting Tyr, Trp, and BCAA availability.	Clinical effects are heterogeneous, and individual amino-acid contributions cannot be separated in combination products.	Investigational adjunct or formulation strategy, not a replacement for complete protein substitution.	[31,42,43]
Unresolved enrichment or delivery strategies	Isolated high-dose enrichment or non-CGMP peptide systems	Individual amino acid/formulation	Potential effects depend on dose, peptide sequence, Phe content, release kinetics, transport, and matrix composition.	Evidence is predominantly mechanistic, preclinical, or product-specific.	Requires residual-Phe analysis, digestibility, pharmacokinetic, and long-term clinical validation.	[10,46]

Note: Categories are descriptive, non-exclusive, and unranked. All indispensable amino acids require adequate provision; their identification as research candidates does not imply that selective enrichment is necessary or clinically beneficial. Individual amino acids, amino-acid mixtures, and delivery systems are different units of consideration and are not treated as directly comparable alternatives. Evidence summaries are narrative descriptions, not formal certainty grades. No numerical ratings, criterion weights, or aggregate scores were assigned. Abbreviations: BCAA, branched-chain amino acid; CGMP, casein glycomacropeptide; LNAA, large neutral amino acid; PAH, phenylalanine hydroxylase; Phe, phenylalanine; Trp, tryptophan; Tyr, tyrosine.

Tyr warrants particular attention because PAH deficiency reduces its endogenous synthesis. This nutritional rationale should be distinguished from the unresolved clinical value of isolated high-dose Tyr supplementation. Trp, Leu, and balanced non-Phe LNAA formulations have mechanistic rationales related to neurotransmitter precursors, anabolic signaling, or transport competition, but these mechanisms do not establish the benefit of selective enrichment. Lys, Thr, Met, His, Ile, and Val remain essential for a complete amino-acid pattern irrespective of whether additional PKU-specific effects have been demonstrated [1,9,24,43]. Formulation objectives determine which questions require further investigation. Studies addressing cerebral amino-acid availability would need to examine LNAA balance and transport-related outcomes; studies addressing protein utilization would need to measure nitrogen retention, amino-acid oxidation, or protein synthesis; and studies addressing practical use would need to evaluate acceptability, tolerance, stability, and adherence. These examples illustrate differences in research objectives, rather than changes in a calculated ranking. No numerical weights were assigned or varied, and no sensitivity analysis was performed. Accordingly, this subsection describes how research questions vary with the intended use; it does not report sensitivity results or demonstrate robustness of a ranking. The principal limitation of this synthesis is the absence of a standardized comparative dataset linking individual amino acids and formulations to all nine domains in Figure 2. Evidence from complete products cannot establish the independent contribution of each amino acid, and mechanistic findings cannot substitute for patient-relevant clinical outcomes. A future quantitative application would require clearly defined alternatives, comparable criterion-specific measurements, explicit handling of missing evidence, transparent weights, and analyses of how uncertainty and alternative weights affect the results. Figure 2 should therefore be interpreted as a conceptual map for organizing evidence and identifying research needs, rather than as a validated selection or dosing algorithm. Because the literature was synthesized narratively without a reported reproducible search and eligibility process, relevant studies may have been omitted, and the balance of cited findings may be affected by selection and citation bias. The descriptions in Table 2 and Figure 2 are not formal assessments of risk of bias or certainty of evidence, and the proposed research directions should be interpreted within these limitations.

## 6. Alternative Delivery Systems Beyond Conventional Free Amino Acids

### 6.1. CGMP: Benefits, Residual Phenylalanine and Limitations

Alternative delivery systems should be evaluated as food matrices rather than merely as nutrient vehicles. Their value depends on the interaction between the ingredient composition, peptide or protein structure, processing history, gastrointestinal behavior, sensory properties, stability, and clinical usability. CGMP is currently the most developed peptide-based option, whereas non-CGMP oligopeptides, engineered low-phenylalanine proteins, encapsulated amino acids, and hybrid systems remain less clinically researched [6,46]. Comparative evaluation should therefore include residual phenylalanine, amino-acid completeness, digestibility, plasma kinetics, oxidation, taste, texture, gastrointestinal tolerance, storage stability, batch reproducibility, and regulatory status.

CGMP is not naturally a complete PKU protein substitute. Its amino-acid sequence is deficient in several indispensable or conditionally important amino acids, including His, Leu, Met, Tyr, and Trp. Commercial CGMP products therefore require supplementation with free amino acids to provide a complete protein-equivalent profile. This hybrid structure is nutritionally important, as the benefits of a peptide matrix may be lost if the added amino acids are delivered as a large, rapidly absorbed bolus. Conversely, an incomplete CGMP formulation cannot support adequate protein synthesis, despite its favorable sensory characteristics [5,6].

Residual Phe represents the most important limitation. Although the Phe concentration of purified CGMP is low, it is not necessarily zero, and residual Phe contributes to the patient’s daily Phe allowance. In a randomized crossover trial, CGMP-based medical food was feasible and nutritionally acceptable, but its use required careful calculation of the total Phe intake and supplementation of limiting amino acids [5]. Pediatric data similarly indicate that the residual Phe content of CGMP products must be considered when interpreting blood-Phe control [47]. For commercial CGMP products, total Phe in each finished-product batch should be assessed against a justified product specification using the validated methods, dose-based reporting, and consistency controls described in the subsection “Free and Total Phenylalanine Analysis and Batch Release”; an intrinsically low-Phe peptide sequence does not establish the Phe content of the commercial formulation.

The clinical evidence does not indicate universal superiority over free-amino-acid medical foods. A systematic review and meta-analysis found limited numbers of trials, small sample sizes, and insufficient evidence for consistent differences in blood Phe, Tyr, or nutritional biomarkers between CGMP-amino-acid and conventional amino-acid products [6]. Thus, CGMP is best regarded as a promising matrix that may improve acceptability, dietary variety, and absorption characteristics, rather than as a complete replacement for amino-acid mixtures. Its future applicability depends on highly purified low-Phe material, complete amino-acid supplementation, controlled peptide digestion, long-term nutritional evaluation and improved patient-centered outcomes.

### 6.2. Oligopeptides and Peptide-Mediated Amino Acid Delivery

Oligopeptide delivery provides a mechanistic alternative to intact proteins and free amino acids. Dietary dipeptides and tripeptides are absorbed principally through the proton-coupled transporter PEPT1, encoded by SLC15A1. They are then hydrolyzed predominantly by intracellular enterocyte peptidases, although selected hydrolysis-resistant peptides may reach portal blood intact [48]. Because PEPT1 accepts a broad range of sequences, peptide-bound amino acids may cross the apical membrane independently of their usual free-amino-acid transporters, potentially reducing competition at this initial absorption step.

The relevance in PKU is plausible, but clinically unproven. A Tyr-, Trp-, or BCAA-containing di- or tripeptide may enter an enterocyte through PEPT1, rather than through an apical neutral-amino-acid transporter. Following hydrolysis, however, the released amino acids remain subject to basolateral export, splanchnic metabolism, systemic distribution, and LAT1 competition at the blood–brain barrier. PEPT1-mediated entry therefore changes the initial absorption route but does not ensure greater systemic retention or cerebral availability. Its physiological effect will depend on the peptide sequence, susceptibility to luminal and intracellular hydrolysis, dose, matrix, and the complete amino-acid pattern [49].

Non-CGMP oligopeptides are investigational candidates for PKU protein substitutes. Their effects on taste, osmolality, and plasma appearance cannot be assumed from peptide structure alone, as protein hydrolysates may be rapidly absorbed, and hydrophobic peptides are often strongly bitter. Any candidate should undergo peptide mapping and molecular-weight characterization, free and total Phe analysis as distinguished in the subsection “Free and Total Phenylalanine Analysis and Batch Release,” gastrointestinal digestion and absorption testing, stability assessment, and batch-level compositional control. Food-derived origin or an initially low-Phe raw material is insufficient without verification in the processed final product [49,50].

CGMP provides the most advanced clinical example of peptide-based PKU nutrition; however, its limitations illustrate the challenges facing other oligopeptides. It contains residual Phe and requires supplementation with rate-limiting amino acids; consequently, the peptide fraction and the free-amino-acid fraction must be evaluated together. For a new oligopeptide product, several traits should be demonstrated, such as at least equivalent Phe control, adequate indispensable-amino-acid delivery, acceptable nitrogen utilization, good gastrointestinal tolerance, and superior or noninferior adherence compared to conventional products [5,9].

The currently available evidence supports consideration of non-CGMP oligopeptides as an investigational platform, not as an established clinical substitute. Their development is justified by the possibility of improving absorption kinetics, palatability, and nitrogen retention, but clinical translation requires studies that compare matched Phe-free amino-acid and peptide formulations using plasma kinetics, urinary nitrogen, protein turnover, gastrointestinal outcomes, metabolic control, and quality of life.

### 6.3. Engineered Low-Phenylalanine Proteins and Hydrolysates

Engineered low-Phe proteins represent a more recent strategy for combining the nutritional and sensory advantages of intact protein with the Phe restriction required in PKU. Recombinant production permits the design of proteins with a defined amino-acid sequence, minimal Phe content and improved digestibility. In principle, the amino-acid composition can be adjusted to correct the limitations of conventional food proteins while maintaining a peptide-rich matrix. This approach differs from CGMP because the protein sequence can be designed rather than selected from a naturally occurring whey fraction.

A study published in 2024 described a Phe-free recombinant nutritional protein produced through biotechnology and evaluated its nutritional potential in PKU-relevant experimental systems [46]. Such products could theoretically provide more physiological gastric and intestinal handling than free amino acids, while avoiding the residual Phe problem associated with many food-derived proteins. They may also offer improved texture, flavor, and incorporation into conventional foods. However, recombinant production introduces its own requirements, including host-cell purification, compositional consistency, allergen assessment, digestibility, regulatory characterization, and total-Phe quantification in the final product using the approach in the subsection “Free and Total Phenylalanine Analysis and Batch Release.”

Protein hydrolysates represent an intermediate compositional form between intact proteins and free-amino-acid mixtures. Enzymatic hydrolysis cleaves peptide bonds, reducing average molecular size and altering solubility, digestibility, and sensory properties. It does not selectively remove phenylalanine: hydrolysates prepared from conventional dairy, soy, or other proteins retain Phe either as free Phe or within peptide sequences unless the source is intrinsically low in Phe or a validated separation step is applied. The residual concentration of Phe consequently depends on the source-protein composition, enzymes and hydrolysis conditions, extent of Phe release, peptide fractionation or adsorption procedures and the analytical method used. Hydrolysates may also vary in their proportions of free amino acids, dipeptides, tripeptides and larger peptides. Degree of hydrolysis alone does not adequately describe this distribution or reliably predict postprandial amino-acid kinetics [51,52]. Commercial hydrolysates should therefore be controlled using finished-product total-Phe specifications and batch-consistency procedures, rather than degree of hydrolysis or free-Phe measurements alone (subsection “Free and Total Phenylalanine Analysis and Batch Release”).

Engineered proteins and protein hydrolysates intended for PKU must be evaluated for limiting amino acids as well as residual Phe. A Phe-free or low-Phe protein may remain nutritionally incomplete if it provides insufficient digestible Lys, Thr, sulfur amino acids, His, Trp, or—specifically in PKU—Tyr. CGMP illustrates this principle: although intrinsically low in Phe, it requires targeted amino-acid supplementation to provide a more complete protein equivalent. A rational and feasible formulation strategy should therefore aim to develop a recombinant or hydrolyzed protein with controlled Phe content, characterize its digestible or metabolically available amino-acid profile, and add the specific amino acids needed to meet an age-appropriate requirement pattern. Complementation should be based on the measured final-product profile and the amount of each amino acid available for metabolism, rather than on a generic fortification formula [46,53].

The principal research gap is the lack of long-term human PKU trials comparing engineered proteins with established free-amino-acid or CGMP products. Short-term digestibility and plasma-kinetic data are necessary but insufficient. Clinical development requires measurement of blood Phe, amino-acid exposure, nitrogen retention, growth, body composition, micronutrient status, gastrointestinal tolerance, sensory acceptability and adherence. Engineered proteins are therefore scientifically promising; however, the technologies required for their development remain under investigation rather than validated alternatives to current medical foods.

### 6.4. Sustained Release, Encapsulated and Hybrid Formulations

Sustained-release formulations aim to modify the rapid appearance of amino acids after ingestion. Conventional free-amino-acid products are absorbed rapidly, since no proteolytic digestion is required, which may result in transient plasma peaks, increased oxidation, and a short period of amino-acid availability for protein synthesis. Prolonged-release technologies use encapsulation, matrix entrapment, modified particles or combinations of polymers and lipids to delay dissolution and intestinal release. The objective is not simply to slow down the absorption, but to generate a plasma profile that preserves total amino-acid exposure while reducing excessive maximum concentrations.

The healthy-volunteer study summarized in Section 3.1 provides preliminary pharmacokinetic evidence for one prolonged-release formulation. It does not establish that controlled-release products reproduce physiological protein delivery or improve long-term PKU outcomes. Physiomimic technology has also been described for taste and odor masking and gastrointestinal release modification [54]; these formulation attributes require separate evaluation from clinical efficacy.

Encapsulation should preserve the chemical stability of the amino acids and provide complete, reproducible release within the absorptive region of the small intestine. Excessively delayed or incomplete release could reduce bioaccessibility, shift part of the dose toward distal intestinal segments, and increase exposure to microbial metabolism. Release should therefore be characterized under physiologically relevant gastric and intestinal pH conditions, in fed and fasted states, across expected gastrointestinal transit times and—when the product is intended for repeated daily use—under multiple-dose conditions. Appropriate evaluation includes in vitro dissolution and stability testing together with plasma *C_max_*, *T_max_*, AUC and, where feasible, the fraction of the administered dose absorbed. Metabolic assessment should be extended to postprandial insulin and glucose, blood urea nitrogen, urinary nitrogen and direct measures of amino-acid oxidation or incorporation when stable-isotope methods are available. A reduced plasma peak accompanied by preserved AUC is consistent with slower systemic appearance; a reduced peak accompanied by substantially lower AUC may instead indicate incomplete delivery, reduced absorption, or altered first-pass metabolism [13,55].

Hybrid protein substitutes combine free amino acids with a peptide or protein fraction, as exemplified by CGMP supplemented with its limiting amino acids. Their theoretical rationale is to combine directly absorbable selected amino acids with amino acids released during digestion of the peptide or protein matrix. This could permit amino-acid-specific tailoring—for example, immediate provision of supplemental Tyr or Leu alongside a compositionally complete protein equivalent—but clinical evidence that such timing improves PKU outcomes is still lacking [56].

Hybrid formulations complicate interpretation because supplemented free amino acids and matrix-bound amino acids follow different initial digestive and transport pathways. Each fraction may also differ in the first-pass metabolism and residual-Phe contribution. Unless the fractions are isotopically labeled or analytically distinguishable, an observed plasma concentration cannot be assigned confidently to the free, peptide-bound, or endogenous amino-acid pool. The presence of a peptide or protein fraction also does not guarantee slower systemic appearance: recent human evidence indicates that supplemented CGMP can produce an overall amino-acid profile similar to a free-amino-acid substitute rather than to slowly digested casein. Superiority must therefore be demonstrated through comparative trials measuring complete concentration–time profiles, total exposure, nitrogen utilization, blood Phe control, safety, nutritional status, and patient-relevant outcomes [5].

Recent clinical work has involved evaluating prolonged-release formulations during pregnancy and in randomized PKU populations, but the evidence remains limited in sample size and duration [57]. The 2026 randomized crossover study on prolonged-release versus standard amino-acid substitute further emphasizes the importance of early-morning Phe fluctuations, although full interpretation requires examination of formulation composition and study endpoints [58]. Sustained release and hybrid products therefore represent promising delivery systems, but their clinical value depends on the demonstrated improvements in nitrogen retention, metabolic stability, tolerability and long-term adherence. Figure 3 provides a schematic comparison of delivery systems, complementing the metabolic characteristics in Table 1A and practical characteristics in Table 1B. These delivery-system comparisons are distinct from the amino-acid roles and enrichment hypotheses summarized in Table 2 and Figure 2. Changes in plasma kinetics or sensory properties should be distinguished from demonstrated improvements in nitrogen retention, metabolic control, or long-term adherence. The healthy-volunteer findings in Section 3.1 do not establish these clinical outcomes.

## 7. Food-Matrix Properties and Technological Performance

Food-matrix and processing characteristics determine whether a nutritionally appropriate amino-acid profile can be translated into an acceptable and effective PKU medical food. Ingredient assessment should include source identity and purity, the degree of hydrolysis, peptide-size and molecular-weight distributions, amino-acid completeness, residual phenylalanine, allergenic potential, and compatibility with the intended food format. Enzymatic hydrolysis might alter solubility, digestibility, rheology, and sensory properties; however, its effects are product-specific, and extensive hydrolysis or peptide fractionation might generate hydrophobic bitter peptides and increase the concentration of small soluble molecules. Larger peptide or protein structures may modify the mouthfeel and digestion kinetics, but slower systemic delivery is not guaranteed, and incomplete digestion, retained Phe-containing sequences, and compositional variability remain possible [59,60]. Consequently, amino-acid analysis should be combined with peptide and molecular-weight profiling, quantitative residual-Phe testing, and assessment of limiting indispensable amino acids, standardized digestibility, and batch consistency.

Solubility, dispersibility, rheology, hygroscopicity, moisture migration, oxidative stability, and other storage-related properties should be evaluated under the intended reconstitution, packaging, and shelf-life conditions. Free-amino-acid protein substitutes commonly have bitter tastes and undesirable odor or aftertaste, whereas peptide- or protein-based matrices may improve sensory acceptability but can introduce allergenic-protein and residual-phenylalanine concerns. Encapsulation, coating, flavor masking, and matrix-based delivery may reduce bitterness and hygroscopicity and improve product stability. However, such processing must preserve the gastrointestinal release and the quantitatively verified delivery of the intended amino acids. Sensory performance is clinically relevant in PKU because poor taste, smell, and texture can reduce adherence, and thereby decrease the actually achieved nutritional exposure [61,62].

Processing and scale-up are integral to formulation performance. Operations such as drying, agglomeration, blending, coating, and extrusion can alter particle-size distribution, morphology, bulk density, flowability, reconstitution, moisture sensitivity, chemical stability, and release behavior. Controlled release and hybrid systems therefore require reproducible manufacturing parameters, predictable dissolution, complete gastrointestinal release, and demonstrated stability during storage. Commercial translation additionally requires standardized raw materials, validated compositional and residual-Phe analyses, allergen and contaminant controls, batch-to-batch manufacturing consistency, economic feasibility, and classification under the applicable medical-food or specialized-nutrition regulatory framework [61,62]. Formulation should be based on the incorporation of evidence on composition, processing characteristics, sensory acceptability, physicochemical and shelf-life stability, digestibility and release, amino-acid kinetics, adherence, and clinically relevant PKU outcomes.

### 7.1. Ingredient and Peptide Source Selection

Ingredient selection should be based on the composition of the source, purity, protein-equivalent completeness, residual phenylalanine, digestibility, allergenicity, sensory behavior, cost, availability, and scale-up potential. Free amino acids provide precise composition and precise control of Phe exposure when assessed as described in the subsection “Free and Total Phenylalanine Analysis and Batch Release,” but they commonly impose bitterness, high osmolality, and reconstitution challenges. CGMP offers a more food-like peptide matrix but remains incomplete without added amino acids and may contain measurable residual phenylalanine. Dairy, plant, microbial, and recombinant proteins provide different peptide profiles, allergenicity profiles, processing requirements, and costs. Engineered low-phenylalanine proteins and defined oligopeptides are promising investigational platforms, but their amino-acid completeness, digestibility, batch reproducibility, and long-term human PKU evidence remain uncertain [6,46].

### 7.2. Enzymatic Hydrolysis and Molecular-Weight Distribution

Enzymatic hydrolysis can be used to adjust peptide size, solubility, viscosity, digestibility, and release behavior. Protease specificity, enzyme-to-substrate ratio, hydrolysis time, temperature, and pH determine the molecular-weight distribution and sequence of the resulting peptides. A greater degree of hydrolysis may improve dispersibility and intestinal accessibility, but can increase bitterness, hygroscopicity, osmolality, and free-amino-acid oxidation. Peptide profiling by size-exclusion chromatography, liquid chromatography, and mass spectrometry should therefore accompany amino-acid analysis. The optimum hydrolysate is not necessarily the most extensively hydrolyzed material; rather, it is the substance that balances complete digestion, acceptable sensory quality, residual-phenylalanine control, and predictable plasma amino-acid appearance [10,54].

### 7.3. Sensory Quality, Flavor Masking, and Gastrointestinal Tolerance

Sensory performance is a determinant of nutritional exposure because poor taste reduces adherence. Free amino acids and short peptides may produce bitterness, sulfur-like or metallic notes, acidity, astringency, and an unpleasant aftertaste. Formulation strategies include flavor masking, bitterness blockers, sweetener and acid balance, aroma modulation, emulsification, microencapsulation, and incorporation into appropriate food matrices. Solubility, viscosity, mouthfeel, reconstitution time, and osmolality must be optimized together, as a taste improvement that increases sedimentation or gastrointestinal discomfort may reduce practical effectiveness. Sensory testing should include trained descriptive analysis and patient-centered acceptability testing, with gastrointestinal symptoms and product use recorded alongside sensory scores [5].

### 7.4. Encapsulation and Controlled-Release Technologies

Encapsulation may separate bitter amino acids from the oral environment and may be used to modify dissolution or release. Lipid, protein, polysaccharide, and hydrocolloid coatings, as well as spray-dried and freeze-dried particles, can be designed to control moisture sensitivity, gastric stability, and intestinal release. Enteric, pH-responsive, and matrix-controlled systems may generate a more prolonged amino-acid appearance, but they must demonstrate complete release, reproducible coating performance, chemical stability, and appropriate intestinal digestion. A slower plasma profile is a pharmacokinetic characteristic, not proof of improved clinical outcome. The healthy-volunteer findings summarized in Section 3.1 cannot establish repeated-dose performance in PKU or be generalized to all encapsulated or hybrid products. Matched-composition PKU studies should assess complete release, plasma kinetics, direct measures of utilization, tolerance, and adherence.

### 7.5. Processing, Scale-Up, and Manufacturing Reproducibility

Blending, dry mixing, spray drying, freeze drying, extrusion, agglomeration, coating, and granulation can alter the particle size, bulk density, powder flow, moisture migration, reconstitution, release kinetics, and storage stability. Process parameters should be linked to amino-acid degradation, residual phenylalanine control, sensory quality, and batch-to-batch composition. Scale-up requires standardized raw materials, validated mixing and coating processes, in-process controls, defined critical quality attributes, and cost evaluation. Formulations that perform well at the laboratory scale may fail during industrial production because of segregation, incomplete coating, poor flow, moisture uptake, or variable release. Reproducibility should therefore be demonstrated across independent batches and realistic storage conditions rather than inferred from a single laboratory preparation [6,46].

### 7.6. Analytical Quality Control, Safety, and Regulatory Requirements

Quality control also includes complete amino-acid profiling, peptide-size distribution, protein-equivalent calculation, digestibility, contaminant and microbial testing, allergen assessment, oxidative stability, and shelf-life. Medical-food classification and labeling remain jurisdiction-specific. The Phe-specific analytical and release approach is detailed below.

#### Free and Total Phenylalanine Analysis and Batch Release

Regulatory requirements should be distinguished from manufacturer-defined release specifications. Commission Delegated Regulation (EU) 2016/128 requires foods for special medical purposes to meet the intended population’s nutritional needs on a scientifically supported basis and prescribes compositional and information requirements [63]. FDA guidance likewise describes the medical-food framework and applicable food-manufacturing and labeling obligations [64]. Neither document specifies a universal numerical residual-Phe limit applicable to all PKU protein substitutes. The numerical example below is therefore a proposed product specification, not a statutory threshold. For each market, any applicable product-specific requirements and the basis of a low-Phe or Phe-free description must be established; a specification should be justified by total Phe at the maximum intended daily intake and the Phe allocation appropriate to the intended population.

Phe analysis should distinguish free Phe in an unhydrolyzed extract from total Phe measured after validated hydrolysis of the complete product, including free, peptide-bound, and protein-bound Phe. Total Phe, expressed as released Phe rather than residue mass, is the appropriate basis for calculating the product contribution to dietary Phe exposure. Total minus free Phe may estimate the bound fraction only when both procedures have comparable validated recoveries, and the uncertainty of the difference is considered. A low free-Phe result alone cannot establish low total Phe.

For free Phe, extract a homogenized test portion without hydrolysis using a validated aqueous or dilute-acid procedure, remove interfering macromolecules as appropriate, and quantify the separated Phe peak. For total Phe, hydrolyze a separate test portion under conditions shown to release Phe quantitatively. Conventional amino-acid analysis uses approximately 6 mol/L HCl at 110 °C for about 24 h, followed by ion-exchange chromatography with post-column ninhydrin detection; the free/total distinction is explicitly described in established amino-acid methods [65]. A food-relevant alternative is acid hydrolysis with pre-column AQC derivatization and reversed-phase UHPLC–UV, as in AOAC 2018.06 [66]. The selected procedure must be validated for the actual PKU formulation and trace-Phe range; validation at ordinary protein concentrations cannot establish suitability near a Phe-free specification.

Validation should address selectivity against abundant amino acids and matrix components, calibration over the intended range, blanks and carry-over, recovery, repeatability, intermediate precision, and method-level detection and quantification limits [67]. Hydrolysis completeness should be assessed using a suitable Phe-containing peptide or protein control and, where appropriate, a hydrolysis time-course. A free-Phe spike alone assesses recovery of added free analyte and does not demonstrate release of bound Phe. Independent sample preparations, not only replicate injections, should be included. Coated or encapsulated products require representative sampling and sample preparation that accesses the whole payload.

Reports should identify the batch, sampling plan, product basis (as sold or reconstituted), extraction and hydrolysis conditions, chromatographic method, recovery information, uncertainty, and limits of detection and quantification after all dilution factors. Report free and total Phe in mg/100 g product, mg per serving, and mg at the maximum intended daily product intake; mg/g protein equivalent may be added for comparisons. Report a result below quantification as “<LOQ” with the numerical LOQ, rather than zero; distinguish detected-but-not-quantifiable results from results below the detection limit. The analytical meaning of a Phe-free claim must be stated and checked against applicable jurisdictional requirements.

A proposed exposure-based specification can be defined as follows: daily product Phe exposure E = C × D, where C is total Phe in mg/g product, and D is the maximum intended product intake in g/day. If A is the individualized daily Phe allowance (mg/day), the product contributes 100 × E/A percent of that allowance. A clinically justified product allocation B (mg/day), defined for the intended population and use, gives a concentration specification Cspec = B/D. Method development should target LOQ × D well below B, allowing room for analytical uncertainty. As an illustrative calculation only, A = 250 mg/day, D = 100 g/day, and B = 5 mg/day give Cspec = 0.05 mg/g (5 mg/100 g); an LOQ of 0.01 mg/g corresponds to 1 mg/day, or 0.4% of A. These are hypothetical design values, not a recommended Phe allowance or universal release limit.

For batch release, use a predefined sampling plan covering relevant locations or units, independent preparations, acceptable analytical controls, and a documented decision rule incorporating measurement uncertainty [67]. One proposed conservative rule for quantified results is C + U ≤ Cspec, where U is expanded uncertainty in the same units, and the coverage factor is reported. Results above this acceptance limit should be held for investigation rather than released under that rule. For <LOQ results, LOQ × D is a useful screening estimate, not a statistical upper confidence bound; compliance still requires a validated low-level decision procedure. Specifications and sampling must address batch heterogeneity, and changes in ingredients or processing require reassessment. A certificate of analysis should state the applicable limit, results, uncertainty where relevant, and release decision.

For commercial hydrolysate- and CGMP-based products, we recommend batch-by-batch confirmation of total Phe in the finished formulation against the predefined specification, using the validated method and uncertainty-based decision rule described above. Supplier certificates should be supported by qualification and periodic verification of incoming materials. Manufacturing controls should address source-protein variability, purification or fractionation performance, mixing uniformity, and carry-over from Phe-containing materials. Batch records should link raw-material lots, processing conditions, sampling locations, analytical results, and the release decision. Results should be trended across batches using predefined alert and action criteria; process drift or an out-of-specification result should trigger investigation and appropriate disposition before release. These are recommended quality-assurance measures, not a claim that the cited regulatory documents prescribe this exact testing schedule [63,64,67].

Previous research identifies residual Phe, amino-acid completeness, palatability, and release reproducibility as critical product attributes. Standardized links between these attributes and clinical performance are still lacking [5,10].

## 8. Research Gaps and Priority Experimental Roadmap

The most important research gap is the absence of integrated, head-to-head studies linking formulation structure to amino-acid utilization and patient-relevant outcomes. Recent studies often examine composition, plasma amino-acid concentrations, sensory acceptability, or phenylalanine control separately, making it difficult to determine whether a slower or more balanced amino-acid profile improves nitrogen retention, protein synthesis, adherence, neurocognitive function, or quality of life. Future studies should compare protein-equivalent doses of free amino acids, CGMP-based mixtures, defined oligopeptides, engineered low-phenylalanine proteins, and controlled-release systems under standardized conditions. Each study should assess residual phenylalanine, peptide composition, digestibility, gastric emptying, plasma amino-acid kinetics, amino-acid oxidation, urea production, nitrogen balance, sensory acceptance, gastrointestinal tolerance, and treatment adherence. Stable-isotope tracer methods are particularly important because plasma concentration alone cannot establish tissue utilization. Longer-term studies should stratify participants by age, phenotype, treatment response, energy intake, exercise, pregnancy, and baseline adherence [11,12]. Age-specific development should prioritize nutritionally complete Phe-free amino-acid formulations during infancy and rapid growth, evaluate palatable CGMP- or peptide–amino-acid hybrids in older children and adolescents when residual Phe can be accommodated, and test flexible prolonged-release or balanced-LNAA strategies in adults. Pregnancy requires tightly controlled Phe exposure and should be evaluated separately [1,47,57]. This coordinated approach would connect formulation characteristics mechanistically and clinically and provide a stronger basis for selecting ingredients and delivery systems for next-generation PKU medical foods. Long-term comparative trials should prespecify patient-reported outcome measures (PROMs) alongside biochemical and nutritional endpoints. Age- and language-appropriate PKU-specific instruments, such as the PKU-QOL questionnaires, can assess the perceived impact of protein-substitute administration and dietary treatment, including self-reported adherence [68]. Repeated assessments should capture taste fatigue, preparation time and complexity, portability, dosing inconvenience, and interference with daily activities, rather than relying on a single taste test. Product-specific items not covered by a validated instrument should be developed with patients and caregivers and evaluated for their measurement properties. Caregiver reports should be distinguished from patient self-reports. PROM findings should be interpreted alongside prescribed-versus-consumed substitute intake, missed doses, and discontinuations with reasons; acceptability or quality-of-life scores alone do not establish adherence. Linking these outcomes to Phe control and nutritional adequacy would help determine whether a formulation offers a sustained practical benefit.

Formulation studies should distinguish participants managed predominantly by dietary restriction, sapropterin responders receiving partial substitution, people in pegvaliase initiation or transition, and stable pegvaliase responders with liberalized diets. Drug treatment, treatment phase, measured Phe tolerance, natural-protein intake, and residual substitute use should be reported. In participants reducing medical foods, relevant outcomes include maintenance of blood Phe control, adequacy of protein and micronutrient intake, and the extent to which a substitute remains necessary, alongside acceptability and adherence.

## 9. Conclusions

Next-generation PKU protein substitutes should be designed as complete food systems in which amino-acid composition, peptide or protein structure, gastrointestinal behavior, sensory quality, physicochemical stability, safety, manufacturability, and clinical usability are optimized together. All indispensable amino acids remain core nutritional requirements, whereas Tyr has the strongest PKU-specific rationale because PAH deficiency limits its endogenous production. Trp, balanced LNAA mixtures, selected BCAAs, oligopeptides, engineered proteins, and controlled-release systems remain promising but require further human PKU studies addressing clinical outcomes. Future progress will depend on integrating food-structure analysis, transporter-informed formulation, stable-isotope studies, sensory engineering, standardized analytical methods, and patient-reported outcome measures alongside biochemical and nutritional endpoints. Repeated assessment of taste fatigue, preparation burden, and treatment acceptability is needed to determine whether formulation improvements are sustained in daily use and translate into better adherence. The contribution of this review is the integration of established nutritional roles with formulation-specific analytical and clinical validation questions. Recognition of Tyr’s nutritional importance is not a new finding. The conceptual framework is intended to support study and product design; it does not establish a quantitatively derived amino-acid ranking, an optimal formulation, or a clinical dosing algorithm. The intended population must also be defined by treatment response: sapropterin may reduce substitute requirements in responsive individuals, while successful pegvaliase therapy may allow withdrawal of Phe-free medical foods. Future product development should therefore address persistent dietary dependence and transitional or partial supplementation needs, rather than assume lifelong complete substitution for every person with PKU.

## Figures and Tables

**Table 1:** (**A**) Nutritional, gastrointestinal, transport, and metabolic comparison of PKU protein-substitute systems. (**B**) Sensory, technological, safety, manufacturing, and clinical-study comparison of PKU protein-substitute systems.

(**A**)																
**Delivery System**	**Structural Form**		**AA Completeness**		**Residual Phe**		**Digestibility/Uptake**		**Intestinal Transport**		**Gastric/Intestinal Release**	**Plasma Kinetics**		**Oxidation/Urea**		**Tissue Availability**
Free-AA mixture	Free L-amino acids		Complete if correctly formulated		Negligible when verified		No proteolysis; direct uptake		AA transporters		Immediate release	Rapid peak; short duration		May increase with imbalance		Requires simultaneous AA availability
CGMP product	Low-Phe peptide plus added AAs		Incomplete native profile		Low but nonzero		Peptide digestion, PEPT1, and AA uptake		PEPT1 plus AA transporters		Matrix-dependent	Intermediate or prolonged		Product-specific		Potentially food-like delivery
Non-CGMP oligopeptides	Defined di-/tripeptides		Must be demonstrated		Sequence-dependent		PEPT1-mediated uptake; intracellular hydrolysis		PEPT1 and AA transporters		Sequence- and matrix-dependent	Intermediate; sequence-dependent		Requires direct measurement		Potentially efficient; unproven in PKU
Hydrolysate	Enzymatically hydrolyzed protein		Source- and process-dependent		Source- and purification-dependent		Peptide and AA absorption		PEPT1 plus AA transporters		Intermediate; MW-dependent	Intermediate and heterogeneous		Product-specific		Depends on limiting AAs
Engineered low-Phe protein	Designed or recombinant protein		Must be demonstrated		Designed to contain low or negligible Phe		Gastric and intestinal proteolysis		Peptide and AA transporters		Protein-like; matrix-dependent	Protein-like; matrix-dependent		Requires human tracer data		Potentially sustained
Sustained-release AA	Encapsulated or matrix-controlled AAs		Can be complete		Negligible if verified		Delayed dissolution; direct uptake		AA transporters		Delayed/controlled	Lower peak; prolonged profile		Hypothesized reduction; unproven		Requires matched tissue studies
Encapsulated system	AA or peptide in carrier		Payload-dependent		Payload-dependent		Carrier release followed by uptake		PEPT1 or AA transporters		pH- or matrix-responsive	Controlled; formulation-specific		Requires direct measurement		Depends on release
Hybrid peptide-AA	Peptide fraction plus free AAs		Can be complete if balanced		Depends on peptide fraction		Combined PEPT1 and AA uptake		PEPT1 plus AA transporters		Mixed rapid/intermediate	Composite profile		Requires matched study		Potentially balanced delivery
Balanced LNAA	Non-Phe: Tyr, Trp, and BCAAs		Not complete unless all indispensable AAs supplied		Usually negligible		Direct AA transport		Shared LNAA systems; LAT1 at BBB		Dose- and matrix-dependent	Formulation-dependent		Requires nitrogen data		Clinical benefit unproven
(**B**)																
**Delivery System**		**Sensory Acceptability**		**GI Tolerance**		**Physicochemical Stability**		**Manufacturability/Scale-Up**		**Safety/Regulation**			**Human PKU Studies and Clinical Use**		**Principal Limitation**	
Free-AA mixture		Often bitter; flavor burden		GI symptoms possible		Good dry stability; hygroscopic		Established blending and filling		Established medical-food class			Established clinical use		Rapid excursions, taste, adherence	
CGMP product		Generally improved; product-dependent		Often improved; residual Phe relevant		Source and batch variability		Commercially feasible		Allergen and medical-food controls			Human comparative evidence; limited duration		Residual Phe and incomplete profile	
Non-CGMP oligopeptides		Potentially improved		Requires tolerance testing		Peptide oxidation and moisture risks		Investigational scale-up		Sequence, purity, allergenicity controls			Mechanistic/preclinical		Limited clinical validation	
Hydrolysate		May improve solubility; bitterness possible		Osmolality may increase		Moisture and oxidation control		Process-sensitive		Source allergenicity and purity			Limited PKU evidence		Variable peptide profile	
Engineered low-Phe protein		Food-like potential		Requires testing		Protein stability and allergenicity		Complex biotechnology scale-up		Regulatory characterization required			Investigational		Manufacturing and clinical validation	
Sustained-release AA		May reduce taste intensity		Release must be complete		Coating and moisture stability		Reproducible coating required		Novel medical-food assessment			Emerging human evidence		Release reproducibility	
Encapsulated system		Potential masking benefit		Carrier tolerance required		Particle and coating stability		Scale-up and validation needed		Carrier safety and labeling			Investigational		Incomplete release or variability	
Hybrid peptide-AA		May combine matrix and precision		Product-specific		Component compatibility		Complex blending and validation		Multiple ingredient controls			Emerging		Difficult dose interpretation	
Balanced LNAA		Taste burden possible		Dose and osmotic effects		AA stability manageable		Blending feasible		Status varies by jurisdiction			Adjunct or investigational		Transport competition is nonselective	

Abbreviations: BCAA, branched-chain amino acid; CGMP, casein glycomacropeptide; LNAA, large neutral amino acid; PAH, phenylalanine hydroxylase; Phe, phenylalanine; Trp, tryptophan; Tyr, tyrosine.

## Data Availability

No new data were created or analyzed in this study. Data sharing is not applicable to this article.
